# Pennsylvania Anatomical Act

**Published:** 1885-02

**Authors:** 


### SELECTIONS.

---

THE PENNSYLVANIA ANATOMICAL ACT.

The medical profession in some of our States, and especially in Illinois, are taking active steps toward obtaining new anatomy acts. In view of this fact, and of the recent passage by the State legislature of Pennsylvania of a new act, it may be worth while to lay before our readers a brief sketch of the law and of its excellent working. In our issue for August 11, 1883, we published the full text of the law as proposed by the anatomists and enacted by the legislature.

It provides a State Anatomical Board composed of the professors and demonstrators of anatomy and of surgery of all incorporated medical and dental schools, and one representative from every such private school. This board shall distribute to each school, in proportion to the number of students in their anatomical and surgical classes, all unclaimed and unknown bodies, which would otherwise be buried at the public expense. Each school reports to the board, at stated times, its number of students as a basis of such distribution. In each county of the State unclaimed bodies may be delivered through the board to any physician in the county who complies with the provisions of the act as to filing a bond. Thus it legalizes dissection throughout the State, and affords facilities for anatomical study to those who could secure no such opportunity, except by leaving their practice and coming to Philadelphia. All bodies not so needed in each county are, at the expense of the board, forwarded for use in the large schools, whose students come from all parts of the State.

This act has only been in operation practically since September, 1883, yet its good results are already apparent. To put such a law in operation in the many counties in the State and in numerous institutions, has required a large amount of labor and much patience and tact. Prejudice has in some cases only slowly yielded, but gradually the operation of the law is being extended over a larger area, and, as the figures will show, it is working most satisfactorily. The voluntary Anatomical Association, which preceded the present board, received and distributed in the year 1879-'80, 243 subjects; in 1880-'81, 222 subjects; in 1881-'82, 302 subjects; and in 1882-'83, 244 subjects. In the year ending April 1, 1884, there were received 283 subjects, but the law had then just begun to be enforced. In the first half of the present year there have been rceeived in all 232 subjects. As the field of operation of the law is being gradually extended, it is fair to estimate that the supply for this year will reach at least a total of 400, and possibly even more. Even this number, however, is far too little for the wants of our great schools. The board, however, are gradually putting the law in working order in counties as yet unreached, and the supply will soon be adequate to all the demands of the schools for properly educating their students.

The Pennsylvania anatomy act has been proved by its working to be the best law in force in this country, and we are glad to learn that the profession in Illinois will endeavor to have substantially the same law passed. We bespeak for them all the help our readers can give them. They have gone to work in the right spirit, and with a systematic effort which deserves and should command success. Such a law is the only means to provide the physician with a knowledge of his fundamental study; and is the surest means of preventing desecration of graveyards—a crime which is so shocking to the moral sense of the entire community.—*The Medical News, Dec. 13th, 1884.*

